# AF-6 is a positive modulator of the PINK1/parkin pathway and is deficient in Parkinson's disease

**DOI:** 10.1093/hmg/ddt058

**Published:** 2013-02-07

**Authors:** Joseph Haskin, Raymonde Szargel, Vered Shani, Lucy N. Mekies, Ruth Rott, Grace G. Y. Lim, Kah-Leong Lim, Rina Bandopadhyay, Herman Wolosker, Simone Engelender

**Affiliations:** 1 Department of Pharmacology and; 2 Department of Biochemistry, The Rappaport Faculty of Medicine and Research Institute, Technion-Israel Institute of Technology, Haifa 31096, Israel; 3 Department of Physiology, Yong Loo Lin School of Medicine, National University of Singapore, 14 Medical Drive, Singapore 117599, Singapore; 4 Reta Lila Weston Institute of Neurological Studies, UCL Institute of Neurology, 1, Wakefield Street, Camden WC1N 1PJ, UK

## Abstract

Parkin E3 ubiquitin-ligase activity and its role in mitochondria homeostasis are thought to play a role in Parkinson's disease (PD). We now report that AF-6 is a novel parkin interacting protein that modulates parkin ubiquitin-ligase activity and mitochondrial roles. Parkin interacts with the AF-6 PDZ region through its C-terminus. This leads to ubiquitination of cytosolic AF-6 and its degradation by the proteasome. On the other hand, endogenous AF-6 robustly increases parkin translocation and ubiquitin-ligase activity at the mitochondria. Mitochondrial AF-6 is not a parkin substrate, but rather co-localizes with parkin and enhances mitochondria degradation through PINK1/parkin-mediated mitophagy. On the other hand, several parkin and PINK1 juvenile disease-mutants are insensitive to AF-6 effects. AF-6 is present in Lewy bodies and its soluble levels are strikingly decreased in the caudate/putamen and substantia nigra of sporadic PD patients, suggesting that decreased AF-6 levels may contribute to the accumulation of dysfunctional mitochondria in the disease. The identification of AF-6 as a positive modulator of parkin translocation to the mitochondria sheds light on the mechanisms involved in PD and underscores AF-6 as a novel target for future therapeutics.

## INTRODUCTION

Parkinson's disease (PD) is characterized by the degeneration of dopaminergic neurons in the substantia nigra and the presence of cytoplasmic inclusions called Lewy bodies (1). Most PD cases are sporadic, but several genes are mutated in hereditary forms of PD, providing clues about the mechanisms involved in the disease (1,2). Mutations in parkin are a common cause of familial PD and are responsible for most of autosomal recessive juvenile parkinsonism; referred here as juvenile PD (3). Although juvenile PD cases have few or absent Lewy bodies (4), parkin is present in the Lewy bodies of sporadic PD cases (5–7).

Parkin is an E3 enzyme (8) that belongs to the class of Ring-IBR-Ring ubiquitin-ligases (9,10). Parkin ubiquitinates different proteins, such as synphilin-1, p38, CDCrel, Pael-R, among others (4). Interaction of parkin with synphilin-1 increases the formation of α-synuclein/synphilin-1 inclusions and may contribute to Lewy body formation (11). Parkin also associates through its C-terminus with the PDZ-containing proteins CASK/LIN-2 and PICK1 (12,13). The interaction of parkin with these PDZ domain proteins targets parkin to the postsynaptic protein complex (12).

Since parkin ubiquitin-ligase activity is decreased in both juvenile and sporadic PD (14–16), it is possible that toxic protein accumulation in PD leads to dopaminergic death. In agreement, the parkin substrate p38 accumulates in parkin knockout mice and in both sporadic and juvenile PD cases (17). In addition, parkin interacts with Paris, a protein that accumulates in PD patients and regulates the levels of the transcription factor PGC-1α (18). Supporting a decreased function of parkin in the disease, nitrosylation of parkin in PD leads to decreased ubiquitin-ligase activity at late time points (14,15). Moreover, phosphorylation by Cdk5 decreases parkin ubiquitin-ligase activity (19), while suppression of Cdk5 activity in mice prevents the dopaminergic toxicity promoted by the PD-related toxin 1-methyl-4-phenyl-1,2,36-tetrahydropyridine (20).

Besides regulating the degradation of substrates, parkin protects neurons from several insults, including α-synuclein toxicity and oxidative stress in neurons (21). Parkin also plays an important role in maintaining mitochondrial function. Suppression or knockout of parkin expression in mice, *Drosophila* or human cells leads to severe mitochondrial dysfunction (22,23). Knockout of PINK1, another protein mutated in familial PD (24), also leads to mitochondrial dysfunction. Strikingly, the effects of PINK1 knockout are reversed by parkin overexpression (25–28), indicating that parkin works downstream of PINK1 in a common pathway involved in maintaining optimal mitochondrial function. Additionally, parkin translocates to damaged mitochondria, in a process mediated by PINK1. Once on the mitochondria, parkin recruits autophagic vesicles leading to subsequent mitophagy (29–32).

The regulation of parkin translocation to the mitochondria, however, is still poorly understood. In addition to PINK1, it is likely that the process requires the concert action of additional partners. We now sought to discover new parkin interacting partners that specifically affect the mitochondrial roles of parkin. We report that parkin interacts with AF-6, which robustly increases its translocation to the mitochondria. AF-6 activates parkin ubiquitin-ligase activity and promotes parkin-mediated mitochondrial clustering and mitophagy. We found that cytosolic AF-6 is also a parkin substrate for the proteasome. AF-6 is present in Lewy bodies and its soluble levels are decreased in sporadic PD brains, suggesting that AF-6 deficiency may contribute to the mitochondrial dysfunction observed in the disease.

## RESULTS

**Figure 1.**
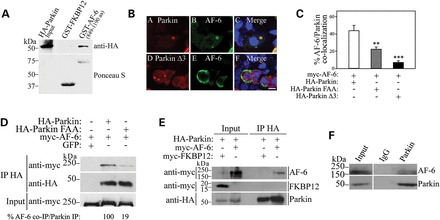
AF-6 interacts with parkin. (**A**) Extracts of HEK293 cells transfected with HA-parkin were incubated with a GST-AF-6 PDZ domain (amino acids 849–1190) or the control GST-FKBP12. Binding was analyzed with an anti-HA antibody. GST fusion proteins used in the experiments were determined by staining the membranes with Ponceau S (lower panel). (**B**) AF-6 co-localizes with inclusions of wild-type parkin but not with a Δ3 C-terminal mutant. HEK293 cells were co-transfected with HA-parkin (wild-type and Δ3 mutant), myc-AF-6, and treated for 16 h with 10 μm lactacystin. Immunocytochemistry was carried out with anti-HA (red, A) and anti-myc antibodies (green, B). Scale bar, 25 μm. (**C**) HEK293 cells were co-transfected with HA-parkin (wild-type, Δ3 and FAA mutants) and myc-AF-6, and treated for 16 h with 10 μm lactacystin. Immunocytochemistry was carried out as in (B). The plot shows the percentage of aggregates in which AF-6 co-localizes with the different parkin constructs. Error bars represent standard error of three to six independent experiments. **, ***Significantly different from wild-type parkin at *P* < 0.01 and at *P* < 0.001, respectively. (**D**) HEK293 cells were co-transfected with myc-AF-6 in the presence of HA-parkin, HA-parkin FAA or GFP. HA-parkin was immunoprecipitated using an anti-HA antibody and samples were analyzed with an anti-myc antibody. The levels of HA-parkin immunoprecipitates were determined using anti-HA antibody (middle panel), while the input levels of myc-AF-6 were determined with an anti-myc antibody (lower panel). (**E**) HEK293 cells were co-transfected with HA-parkin and myc-AF-6 or myc-FKBP12 as control. HA-parkin was immunoprecipitated using anti-HA and samples were analyzed with an anti-myc antibody. Co-immunoprecipitation of myc-AF-6 was observed with parkin but not with the control protein FKBP12 (upper and middle panels). The levels of HA-immunoprecipitates were revealed using an anti-HA antibody (lower panel). (**F**) Endogenous parkin was immunoprecipitated from rat brain lysate using an anti-parkin antibody and co-immunoprecipitation was revealed with an anti-AF-6 antibody. Co-immunoprecipitation of AF-6 was not observed in control coupled to IgG. The figures are representative of three independent experiments.

AF-6 is expressed in the brain and is important for dendritic spine plasticity (33). Similar to the parkin-interacting proteins CASK and PICK1 (12,13), AF-6 contains a PDZ domain and its levels are correlated with parkin in certain types of cancers (34). This led us to investigate a possible direct interaction of parkin with AF-6. For this purpose, we incubated lysates of HEK293 cells transfected with HA-parkin with recombinant glutatione S-transferase (GST)-AF-6 (amino acids 849–1190), encompassing the AF-6 PDZ domain. We observed that parkin was pulled-down by AF-6 but not by a control protein (Fig. 1A), indicating that parkin and AF-6 interact *in vitro*.

Using an algorithm to predict the interaction with PDZ domains (35), we found that the last three amino acids of parkin likely interact with the AF-6 PDZ region. To test the *in silico* prediction, we generated a parkin construct devoid of the last three amino acids (parkin Δ3). However, since the deletion of parkin's last amino acids promotes massive aggregation of parkin and inclusion formation *per se* (36), we carried out immunocytochemistry experiments to evaluate the interaction with AF-6. Since wild-type parkin does not form inclusion unless under conditions of proteasome inhibition (19,37), the experiments were carried out in the presence of lactacystin to allow a comparable amount of inclusion formation among the different parkin constructs. We found that AF-6 co-aggregates with inclusions of wild-type parkin upon proteasome inhibition but not with inclusions of parkin Δ3 (Fig. 1B and C). To further confirm that the C-terminus of parkin is responsible for the interaction with AF-6, we mutated the last two amino acid residues of parkin from FDV to FAA and found that in the presence of lactacystin, AF-6 co-aggregates to a smaller extent with parkin phenylalanine, alanine, alanine (FAA) in comparison to wild-type parkin (Fig. 1C).

Since parkin FAA did not aggregate as much as parkin Δ3, we were able to carry out a series of biochemical experiments in order to confirm the role of parkin's C-terminal in the interaction with AF-6. We investigated the ability of AF-6 to interact with parkin FAA by carrying out co-immunoprecipitation experiments from HEK293-transfected cells. We found that AF-6 co-immunoprecipitated with wild-type parkin but much less efficiently with parkin FAA (Fig. 1D), further supporting the role of the parkin C-terminal region in the interaction. AF-6 also did not co-immunoprecipitate with beads alone (Fig. 1D), indicating the specificity of the interactions. As an additional control, we found that the unrelated protein FKBP12 does not co-immunoprecipitate with parkin (Fig. 1E), giving further indication that the association with AF-6 is specific. Taken together, the results indicate that parkin–AF-6 interaction is mostly mediated by interaction of parkin C-terminal with the PDZ of AF-6.

Next, we carried out co-immunoprecipitation experiments using rat brain tissues and found that endogenous AF-6 co-immunoprecipitates with parkin but not with the control IgG (Fig. 1F), supporting the *in vivo* interaction between parkin and AF-6.

**Figure 2.**
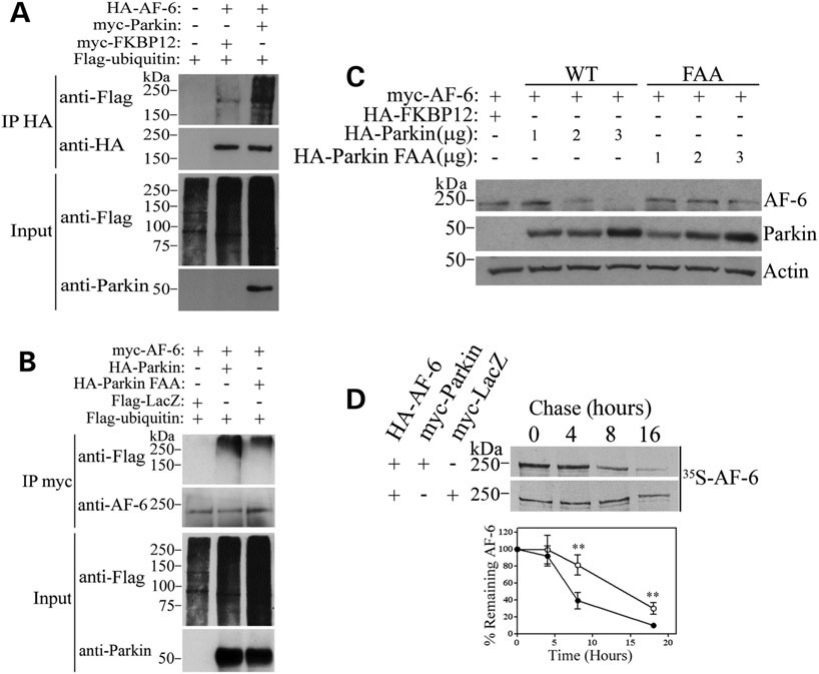
Cytosolic AF-6 is a parkin substrate. (**A**) HEK293 cells were co-transfected with HA-AF-6, Flag-ubiquitin, in the presence of myc-parkin or myc-FKBP12. Cells were incubated for 12 h with 10 μm lactacystin, and HA-AF-6 was immunoprecipitated with an anti-HA antibody. Ubiquitination of AF-6 was detected with an anti-Flag antibody. The second panel shows the levels of immunoprecipitated AF-6 using an anti-HA antibody. Third and fourth panels show the input consisting of total ubiquitinated proteins and parkin, respectively. (**B**) HEK293 cells were co-transfected with myc-AF-6 and Flag-ubiquitin, in the presence of HA-parkin, HA-parkin FAA or Flag-LacZ. Cells were incubated for 16 h with 10 μm lactacystin, and myc-AF-6 was immunoprecipitated with an anti-myc antibody. Ubiquitination of AF-6 was detected with an anti-Flag antibody. The second panel shows the levels of immunoprecipitated AF-6 using an anti-AF-6 antibody. Third and fourth panels show the input consisting of total ubiquitinated proteins and parkin, respectively. (**C**) Parkin lowers the steady-state levels of AF-6. HEK293 cells were transfected with myc-AF-6 and increasing amounts of HA-parkin or HA-parkin FAA. The steady-state levels of myc-AF-6 from total cell lysates were detected by western blot using an anti-myc antibody (upper panel). The expression levels of parkin (WT and FAA) were determined using an anti-parkin antibody. Loading control was monitored with an anti-actin antibody (lower panel). (**D**) Parkin decreases the half-life of cytosolic AF-6. HEK293 cells transfected with HA-AF-6 and myc-parkin were chased for the indicated time points, and HA-AF-6 was immunoprecipitated using an anti-HA antibody. Immunoprecipitates were analyzed by SDS–PAGE and autoradiography (upper panels). Graph shows the quantification of remaining AF-6 in the presence of LacZ (open circle) or parkin (filled circle) at indicated time points. Error bars represent standard error of three independent experiments. **Significantly different from control at *P* < 0.01.

In order to determine if AF-6 is a parkin substrate, we carried out *in vivo* ubiquitination experiments where we immunoprecipitated AF-6 and found that parkin promotes its ubiquitination in a specific manner (Fig. 2A). In agreement, parkin FAA is less efficient than wild-type parkin to ubiquitinate AF-6 (Fig. 2B). We next investigated whether the ubiquitination by parkin leads to AF-6 degradation, and found that increasing amounts of wild-type parkin decrease AF-6 steady-state levels while parkin FAA promotes a smaller decrease of AF-6 steady-state levels (Fig. 2C). Pulse-chase analysis of ^35^S-labeled immunoprecipitated AF-6 revealed that parkin decreases AF-6 half-life (Fig. 2D), indicating that ubiquitination by parkin leads to AF-6 degradation.

**Figure 3.**
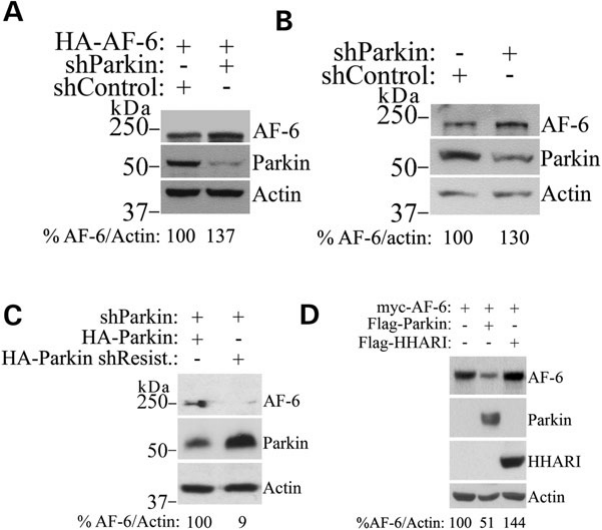
Endogenous parkin regulates AF-6 levels. (**A**) Knockdown of parkin increase the steady-state levels of overexpressed AF-6. HEK293 cells were transfected with HA-AF-6 in the presence of shRNA control or shRNA to parkin. HA-AF-6 from total cell lysates was detected using an anti-HA antibody (upper panel). The presence of parkin was monitored with an anti-parkin antibody (middle panel). Loading control was monitored with an anti-actin antibody (lower panel). (**B**) Knockdown of parkin increases the steady-state levels of endogenous AF-6. HEK293 cells were transfected with shRNA control or shRNA to parkin. AF-6 from total cell lysates was detected using an anti-AF-6 antibody (upper panel). The presence of parkin was monitored with an anti-parkin antibody (middle panel). Loading control was monitored with an anti-actin antibody (lower panel). (**C**) Transfection of shRNA-resistant HA-parkin rescued the effects of shRNA-mediated parkin knockdown. HEK293 cells were transfected with shRNA to parkin, in the presence of 2 μg HA-parkin or shRNA-resistant HA-parkin. AF-6 from total cell lysates was detected using an anti-AF-6 antibody (upper panel). Parkin was detected with an anti-HA antibody (middle panel), while loading control was monitored with an anti-actin antibody (lower panel). (**D**) AF-6 steady-state levels are not affected by the Ring-IBR-Ring E3 ubiquitin-ligase HHARI. HEK293 cells were transfected with myc-AF-6 in the presence of Flag-parkin or Flag-HHARI. The second and third panels show the levels of parkin and HHARI with their specific antibodies. Loading control was monitored with an anti-actin antibody (fourth panel). Percent of AF-6 levels corrected by the levels of actin is shown at the bottom of each figure.

To further determine the relevance of AF-6 degradation mediated by parkin, we carried out experiments in which we knocked down the expression of endogenous parkin using shRNA. We found that shRNA to parkin increases the steady-state levels of overexpressed (Fig. 3A) as well as of endogenous AF-6 (Fig. 3B), indicating that AF-6 may represent an authentic substrate of parkin. Furthermore, transfection of shRNA-resistant parkin rescued the shRNA effects on AF-6 levels, indicating the specificity of the shRNA used (Fig. 3C). Moreover, an additional E3 ubiquitin-ligase that belongs to the group of parkin Ring-IBR-Ring ligases, HHARI (38), does not decrease AF-6 steady-state levels (Fig. 3D), confirming the specificity of AF-6 ubiquitination and degradation by parkin.

**Figure 4.**
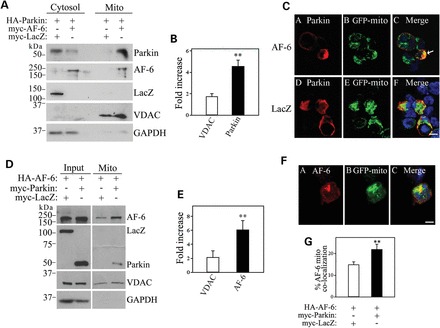
AF-6 promotes parkin translocation to the mitochondria. (**A**) HEK293 cells were transfected with HA-parkin in the presence of myc-AF-6 or myc-LacZ, and lysates were processed to isolate cytosolic and mitochondrial fractions. Translocation of parkin to the mitochondria by AF-6 was determined with an anti-parkin antibody (first panel). The second panel shows the presence of myc-AF-6 in the mitochondrial fraction as well (anti-AF-6 antibody). The third panel shows that myc-LacZ does not translocate to the mitochondria in the presence of parkin (anti-myc antibody). The purity of cytosolic and mitochondrial fractions was determined with anti-GAPDH and anti-VDAC, respectively. (**B**) HEK293 cells were transfected with HA-parkin in the presence of myc-AF-6 or myc-LacZ. Cell lysates were processed as in (A). The presence of parkin and VDAC in mitochondrial fraction was determined by ImageMaster. The graph shows the fold increase in mitochondrial parkin and VDAC promoted by AF-6 when compared with LacZ control. Error bars represent standard error of four independent experiments. (**C**) SH-SY5Y cells were transfected with HA-parkin and the mitochondrial marker GFP-mito, in the presence of either myc-AF-6 or myc-LacZ. Immunocytochemistry and confocal microscopy were carried out with an anti-HA antibody to reveal HA-parkin (red, A and D) and GFP-mito fluorescence (green, B and E). Scale bar, 25 μm. (**D**) HEK293 cells were transfected with HA-AF-6 in the presence of 1 μg myc-parkin or myc-LacZ, and lysates were processed to isolate mitochondrial fractions (right panels). Total inputs are shown in left panels. Translocation of AF-6 to the mitochondria by parkin was determined with an anti-AF-6 antibody (first panel). The second panel shows the presence of myc-parkin but not myc-LacZ in the mitochondrial fraction. The loading of mitochondrial fractions was determined with anti-VDAC. (**E**) HEK293 cells were transfected with HA-AF-6 in the presence of myc-parkin or myc-LacZ. Cell lysates were processed as in (A). The graph shows the fold increase in mitochondrial AF-6 and VDAC promoted by parkin when compared with LacZ control. Error bars represent standard error of three independent experiments. (**F**) SH-SY5Y cells were transfected with HA-parkin, myc-AF-6 and GFP-mito. Immunocytochemistry was carried out with anti-myc antibody. Confocal microscopy pictures depict both myc-AF-6 (red, A) and GFP-mito (green, B). Scale bar, 25 μm. (**G**) SH-SY5Y cells were transfected with HA-AF-6 and GFP-mito, and also with myc-parkin or myc-LacZ. Immunocytochemistry was carried out with anti-HA antibody and analyzed as in F. The graph shows the increase in mitochondrial AF-6 promoted by parkin when compared to LacZ control. Error bars represent standard error of three independent experiments. **Significantly different from VDAC at *P* < 0.01 (B and E). **Significantly different from control at *P* < 0.01 (G).

Since parkin translocation to the mitochondria is thought to play a role in PD, we investigated whether AF-6 modulates this process. For this, we purified mitochondrial fractions of transfected cells and observed that AF-6 increases the levels of parkin at the mitochondria by almost 5-fold (Fig. 4A and B). AF-6 had a much less effect on the levels of the mitochondrial marker voltage-dependent anion channel (VDAC), indicating that the increase in mitochondrial parkin is not due to higher mitochondria biogenesis (Fig. 4A and B). Furthermore, we found that AF-6 co-translocates along with parkin to the mitochondria (Fig. 4A), further supporting their biochemical interaction. The effect of AF-6 in increasing parkin translocation to the mitochondria was also observed in immunocytochemistry experiments, as shown by the increased co-localization of parkin with the mitochondrial marker GFP-mito promoted by AF-6 when compared with a control protein (Fig. 4C).

We also carried out the converse experiments where we investigated the effect of parkin on AF-6 translocation to the mitochondria. We found that parkin transfection, at levels that do not degrade cytosolic AF-6 (1 μg, Fig. 2C), increases the levels of AF-6 at the mitochondria (Fig. 4D and E). Moreover, AF-6 partly co-localizes with GFP-mito and this co-localization is increased in the presence of parkin (Fig. 4F and G), confirming the specific translocation of AF-6 to the mitochondria. Thus, in contrast to the cytosolic AF-6, mitochondria-associated AF-6 is not degraded by parkin.

**Figure 5.**
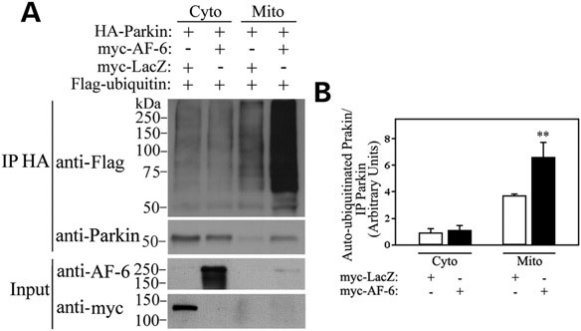
AF-6 activates parkin ubiquitin-ligase activity at the mitochondria. (**A**) HEK293 cells were transfected with HA-parkin and Flag-ubiquitin, in the presence of either myc-AF-6 or myc-LacZ. Lysates were fractionated into cytosolic and mitochondrial fractions, and parkin was immunoprecipitated from each fraction using an anti-HA antibody. Parkin auto-ubiquitination was determined using an anti-Flag antibody (upper panel). The levels of HA-parkin immunoprecipitates were determined using an anti-HA antibody (second panel). The levels of AF-6 and LacZ in the total cells lysates were determined using an anti-myc antibody (third and fourth panels). (**B**) HEK293 cells were transfected and processed as in (A). The levels of auto-ubiquitinated HA-parkin and total immunoprecipitated HA-parkin were determined by ImageMaster. The graph shows the quantification of auto-ubiquitinated HA-parkin relative to immunoprecipitated HA-parkin from cytosolic and mitochondrial fractions. Error bars represent standard error of three independent experiments. **Significantly different from control at *P* < 0.01.

We next investigated how AF-6 interferes with parkin ubiquitin-ligase activity at the mitochondria. We isolated cytosolic and mitochondrial fractions from transfected cells, immunoprecipitated parkin from both fractions, and determined parkin ubiquitin-ligase activity by its auto-ubiquitination levels relative to immunoprecipitated parkin. We found that AF-6 robustly increases the amount of auto-ubiquitinated parkin in the mitochondria compared with LacZ (Fig. 5A and B). Even though the translocation of parkin to the mitochondria *per se* leads to an increase in its ubiquitin-ligase activity (Fig. 5A and B) and (29), AF-6 clearly potentiates this effect, indicating a direct effect on parkin ubiquitin-ligase activity at the mitochondria.

**Figure 6.**
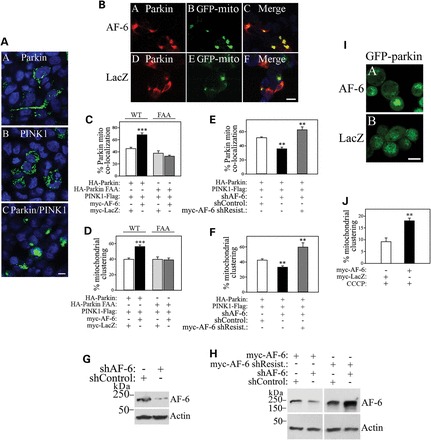
AF-6 increases mitochondrial clustering. (**A**) SH-SY5Y cells were transfected with GFP-mito, in the presence of myc-parkin (A), PINK1-HA (B) and myc-parkin together with PINK1-HA (C). Confocal microscopy depicts GFP-mito and DAPI fluorescence. Scale bar, 25 μm. (**B**) SH-SY5Y cells were transfected with GFP-mito, HA-parkin, PINK1-Flag, in the presence of either myc-AF-6 or myc-LacZ. Immunocytochemistry and confocal microscopy were carried out with anti-HA (red, A and D) and GFP-mito fluorescence (green, B and E). Scale bar, 25 μm. (**C** and **D**) Plots show the quantification of AF-6-mediated increase in parkin translocation (C) and mitochondrial clustering (D) relative to LacZ. Plots also show the inability of AF-6 to increase parkin FAA translocation (C) and mitochondrial clustering (D). SH-SY5Y cells were transfected, processed and analyzed as in (B), in the presence of HA-parkin wild-type or FAA. Error bars represent standard error of 8–12 independent experiments. ***Significantly different from LacZ control at *P* < 0.001. (**E** and **F**) SH-SY5Y cells were transfected with GFP-mito, HA-parkin, PINK1-Flag, with either shRNA control or shRNA to AF-6. Plots show the quantification of parkin translocation (E) and mitochondrial clustering (F) upon AF-6 knockdown. Transfection of shRNA-resistant myc-AF-6 rescued the effects of shRNA-mediated AF-6 knockdown on parkin translocation (E) and mitochondrial clustering (F) (gray bars). Error bars represent standard error of four to seven independent experiments. **Significantly different from shRNA control at *P* < 0.01. (**G**) SH-SY5Y cells were transfected with shRNA to AF-6 or shRNA control. The presence of AF-6 in total cell lysates was detected using an anti-AF-6 antibody (upper panel). Loading control was monitored with an anti-actin antibody (lower panel). (**H**) SH-SY5Y cells were transfected with shRNA to AF-6 or shRNA control, in the presence of myc-AF-6 or or shRNA-resistant myc-AF-6. The presence of AF-6 in total cell lysates was detected using anti-AF-6 antibody (upper panel). Loading control was monitored with an anti-actin antibody (lower panel). (**I**) HeLa cells stably expressing GFP-parkin were transfected with myc-AF-6 or myc-LacZ. Cells were treated with 10 μm CCCP for 1 h and analyzed by confocal microscopy to reveal GFP-parkin and its clustering at the mitochondria upon CCCP treatment. Scale bar, 25 μm. (**J**) The graph shows the quantification of AF-6-mediated increase in mitochondrial clustering assessed by GFP-parkin fluorescence. HeLa cells stably expressing GFP-parkin were transfected and processed as in I. Error bars represent standard error of three independent experiments. **Significantly different from control at *P* < 0.01.

The activation of PINK1/parkin pathway promotes the clustering of mitochondria, which is subsequently degraded by mitophagy (32,39). In agreement, we found that parkin and PINK1 overexpression promotes massive clustering of mitochondria (Fig. 6A). Next, we tested the effect of AF-6 on the PINK1/parkin pathway. We found that in addition to increase parkin translocation (Fig. 6B and C), AF-6 significantly increased the mitochondrial clustering mediated by parkin and PINK1 (Fig. 6B and D). Additionally, AF-6 was ineffective in increasing the translocation of parkin FAA to the mitochondria as well as in increasing mitochondrial clustering in the presence of this mutant (Fig. 6C and D), a result compatible with the decreased ability of AF-6 to interact with parkin FAA (Fig. 1D). Moreover, we observed similar effects of AF-6 on translocation of parkin and mitochondrial clustering in HEK293 cells and HeLa cells (data not shown).

To confirm the role of endogenous AF-6, we employed an shRNA strategy. Knockdown of endogenous AF-6 decreased both parkin translocation and mitochondrial clustering (Fig. 6E and F). To rule off-target effects of the shRNA to AF-6, we examined whether the reduced parkin translocation and mitochondrial clustering could be rescued by full-length shRNA-resistant AF-6 (Fig. 6G and H). Indeed, expression of shRNA-resistant AF-6 rescued parkin translocation and mitochondrial clustering in the presence of the shRNA (Fig. 6E and F), suggesting that AF-6 works as an endogenous regulator of the PINK1/parkin pathway.

Depolarization by the ionophore carbonyl cyanide m-chlorophenyl hydrazone (CCCP) promotes the translocation of parkin to the mitochondria (30,31). To determine whether AF-6 also increases the translocation of parkin to depolarized mitochondria, we transfected HeLa cells stably expressing GFP-parkin with AF-6. We found that AF-6 significantly increases the translocation of parkin even to mitochondria depolarized by CCCP (Fig. 6I and J), indicating that the two processes are additive.

**Figure 7.**
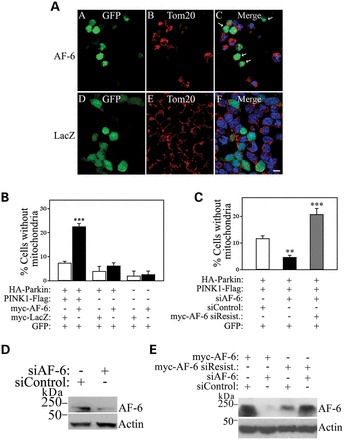
AF-6 increases PINK1/parkin-mediated mitophagy. (**A**) SH-SY5Y cells were transfected with HA-parkin, PINK1-Flag and GFP, and with either myc-AF-6 (upper panel) or myc-LacZ (lower panel). Immunocytochemistry was carried out with anti-Tom20 (red, B and E) and GFP fluorescence (green, A and D). Arrows in (C) indicate transfected cells that lack mitochondria. Scale bar, 25 μm. (**B**) The graph shows the quantification of transfected cells lacking mitochondria. SH-SY5Y cells were transfected and analyzed as in (A). Error bars represent standard error of four independent experiments. (**C**) SH-SY5Y cells were transfected with HA-parkin, PINK1-Flag and GFP, either with siRNA control or siRNA to AF-6. Transfection of siRNA-resistant myc-AF-6 rescued the effects of siRNA-mediated AF-6 knockdown (gray bar). Error bars represent standard error of three to six independent experiments. (**D**) SH-SY5Y cells were transfected with siRNA to AF-6 or siRNA control. The presence of AF-6 in total cell lysates was detected using an anti-AF-6 antibody (upper panel). Loading control was monitored with an anti-actin antibody (lower panel). (**E**) SH-SY5Y cells were transfected with siRNA to AF-6 or siRNA control, in the presence of myc-AF-6 or siRNA-resistant myc-AF-6. The presence of AF-6 in total cell lysates was detected using an anti-AF-6 antibody (upper panel). Loading control was monitored with an anti-actin antibody (lower panel). **, ***Significantly different from control at *P* < 0.01 and 0.001, respectively.

We next examined whether AF-6 affects mitophagy. We found that AF-6 increases the amount of PINK1/parkin-mediated mitophagy, as shown by the lower number of mitochondria in cells co-transfected with AF-6 when compared with cells co-transfected with control LacZ, as assessed by the mitochondrial marker Tom-20 (Fig. 7A and B). The effect of AF-6 on mitophagy was confirmed as siRNA-mediated AF-6 knockdown decreases PINK1/parkin-dependent mitophagy (Fig. 7C). Moreover, the decrease in mitophagy observed when AF-6 is knocked down is specific since the transfection of siRNA-resistant recombinant AF-6 rescues this effect (Fig. 7C–E). Therefore, AF-6 seems to fully activate the PINK1/parkin pathway, from the initial steps of parkin translocation through mitochondrial degradation by mitophagy.

**Figure 8.**
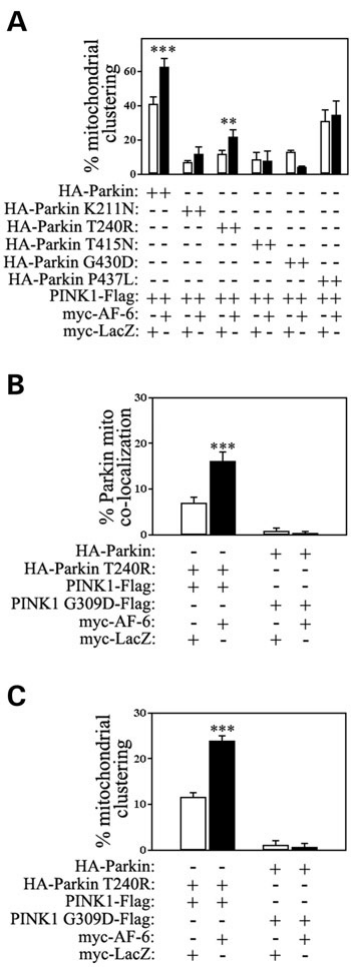
Effects of AF-6 on parkin and PINK1 familial disease-mutation deficiencies. (**A**) SH-SY5Y cells were transfected with the indicated Flag-parkin constructs, wild-type PINK1-HA and GFP-mito, and with either myc-AF-6 or myc-LacZ. Mitochondrial clustering was determined by analyzing GFP-mito using confocal microscopy. Error bars represent standard error of three independent experiments. **, ***Significantly different from LacZ control at *P* < 0.01 and 0.001, respectively. (**B**) SH-SY5Y cells were transfected with HA-parkin (wild-type or T240R), PINK1-Flag (wild-type or G309D) and GFP-mito, and with either myc-AF-6 or myc-LacZ. Parkin localization at the mitochondria was analyzed by immunocytochemistry with anti-HA and co-localization with GFP-mito analyzed by confocal microscopy. The graph shows the quantification of AF-6 effect in the translocation of parkin T240R. AF-6 did not rescue the deficiency in wild-type parkin translocation in the presence of PINK1 G309D disease-mutant. Error bars represent standard error of four independent experiments. ***Significantly different from LacZ control at *P* < 0.001. (**C**) Graph shows the quantification of AF-6 effect in the mitochondrial clustering induced by parkin T240R. AF-6 did not rescue the mitochondrial clustering deficiency in the presence of G309D mutant. SH-SY5Y cells were transfected and analyzed as in (B). Error bars represent standard error of four independent experiments. ***Significantly different from control at *P* < 0.001.

Some parkin juvenile disease-mutants are unable to translocate to the mitochondria (29,40), while PINK1 mutations disrupt parkin recruitment to the mitochondria (31). We next sought to investigate whether AF-6 could overcome the defects of parkin and PINK1 mutants. Among the five different parkin mutants analyzed, AF-6 was only able to partially increase the translocation of the T240R mutant (Fig. 8A–C). Although AF-6 also partially increases the mitochondrial clustering with T240R (Fig. 8C), it fails to increase the mitophagy with this mutant (data not shown). Furthermore, AF-6 does not increase parkin translocation and mitochondrial clustering when in the presence of the PINK1 G309D mutant (Fig. 8B and C), suggesting that AF-6 requires intact PINK1 to promote the mitochondrial translocation of parkin.

**Figure 9.**
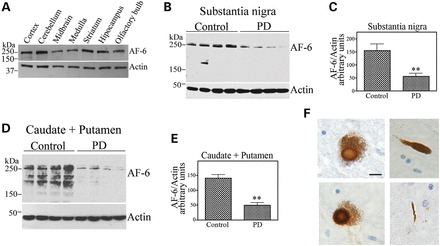
AF-6 is decreased in PD patients and aggregates into Lewy bodies. (**A**) Homogenates prepared from different rat brain regions were probed with an anti-AF-6 antibody (upper panel). Loading control was monitored with an anti-actin antibody (lower panel). (**B**) Homogenates prepared from control and PD substantia nigra tissues were probed with an anti-AF-6 antibody (upper panel). Loading control was monitored with an anti-actin antibody (lower panel). (**C**) The graph represents the average of AF-6 levels in the substantia nigra shown in (B). Levels of proteins were determined by ImageMaster analysis and AF-6 were normalized relative to actin. Error bars represent standard error of four different patients. **Significantly different from control at *P* < 0.01. (**D**) Homogenates prepared from control and PD caudate and putamen tissues were probed with an anti-AF-6 antibody (upper panel). Loading control was monitored with an anti-actin antibody (lower panel). (**E**) The Graph represents the average of AF-6 levels in the caudate and putamen shown in (D). Levels of proteins were determined by ImageMaster analysis and AF-6 were normalized relative to actin. Error bars represent standard error of four different patients. **Significantly different from control at *P* < 0.01. (**F**) Immunohistochemistry analysis of midbrain sections incubated with a purified anti-AF-6 antibody. Lewy bodies containing AF-6 are shown in sections from two distinct PD cases (left panels). Right panels show the presence of AF-6 in Lewy neurites. Scale bar is 20 μm.

We also characterized the relevance of AF-6 to PD by analyzing its expression in the brain of rats and patients. We found that AF-6 is widely expressed in the rat brain, including the midbrain that contains the substantia nigra and is affected in PD (Fig. 9A). We next investigated the levels of AF-6 in PD brains. We found that the steady-state levels of soluble AF-6 are significantly lower in the caudate/putamen as well as in the substantia nigra of PD patients (Fig. 9B–E). We also detected AF-6 in ∼25% of mature Lewy bodies and in occasional Lewy neurites in the substantia nigra of the four PD brains analyzed (Fig. 9F).

## DISCUSSION

We found that parkin interacts with the PDZ-containing protein AF-6 *in vitro* and *in vivo*. Parkin promotes the ubiquitination and degradation of cytosolic AF-6. Knockdown of parkin leads to accumulation of endogenous AF-6, indicating that cytosolic AF-6 is a parkin substrate. On the other hand, the interaction of AF-6 with parkin has a different role at the mitochondria where parkin does not degrade AF-6. We found that endogenous AF-6 increases the translocation of parkin to the mitochondria, enhancing its ubiquitin-ligase activity, and promoting mitochondrial clustering and mitophagy. Our data indicate that AF-6 is a positive modulator of parkin recruitment and activity at the mitochondria via the parkin/PINK1 pathway. The substantial decrease in soluble AF-6 in sporadic PD brain tissues indicates that AF-6 deficiency may contribute to the disruption of mitochondrial homeostasis thought to occur in PD.

AF-6 is important for cell–cell contact by the formation and maintenance of adherent and tight junctions (41,42). Nevertheless, AF-6 translocates to the nucleus in a growth factor-regulated and cell cycle dependent fashion, suggesting that AF-6 may also affect cellular growth (43). Moreover, AF-6 also regulates activity-dependent dendritic spine plasticity in cortical neurons (33). Thus, our findings of AF-6 translocation to the mitochondria and its ability to positively modulate the parkin/PINK1 pathway fits with the multiple roles of AF-6 in signal transduction.

The interaction of parkin with the PDZ domain of AF-6 mostly depends on its C-terminal region which is in accordance with classical PDZ interaction mechanisms (44). In agreement, deletion of parkin last three amino acid residues prevented its co-localization with AF-6. Since the deletion of these last three amino acids promotes a drastic aggregation of parkin, we further confirmed the specificity of the interaction by mutating parkin's last two amino acids (parkin FAA), which was less prone to aggregation. Parkin FAA exhibited reduced interaction and co-localization with AF-6, and promoted less ubiquitination and degradation of AF-6 compared with wild-type parkin. Moreover, AF-6 did not increase parkin FAA translocation to the mitochondria and mitochondrial clustering, further supporting the notion that the interaction of AF-6 with parkin requires the interaction between parkin C-terminus and the AF-6 PDZ domain. Parkin also interacts with the PDZ-containing proteins, CASK/LIN-2 and PICK1 (12,13), which are known as synaptic proteins. Their interaction implies that parkin has a role in regulating some aspects of the synaptic transmission (45,46). In this framework, we hypothesize that the interaction of parkin with different PDZ-containing proteins may represent a *modus operandi*, allowing it to carry out various functions at different cell compartments.

The translocation of parkin to the mitochondria activates its E3 ubiquitin-ligase activity (29). In agreement, we found that parkin is indeed more active at the mitochondria and that AF-6 increases even more its activity at this organelle. Since the interaction of parkin *N*-terminal ubiquitin-like domain with the C-terminus prevents its activation (47), it is possible that binding to the mitochondria weakens the auto-inhibition of parkin by altering the interaction between the *N*- and C-terminal regions. In this framework, binding of AF-6 to the C-terminus of parkin seems to be required to fully activate parkin by relieving its auto-inhibition at the mitochondria. Moreover, as proteasomal activation at the mitochondria is an essential step prior to mitophagy (48,49), activation of parkin ubiquitin-ligase activity at the mitochondria may represent the mechanism by which AF-6 activates mitophagy as well. Parkin increases AF-6 translocation and levels of AF-6 at the mitochondria. For these experiments, we used a low parkin concentration that does not degrade cytosolic AF-6. It is not clear why mitochondrial AF-6 is not degraded by mitochondrial parkin, while cytosolic AF-6 is a parkin substrate. One possibility is that binding to the mitochondria shields AF-6 ubiquitination sites or that parkin ligase activity at the mitochondria is diverted toward more effective substrates including a plethora of outer membrane mitochondrial proteins (48,50,51).

A few proteins were shown to influence parkin/PINK1 pathway. For instance, Ambra1 affects the mitochondrial clearance but not the translocation of parkin (52). Yet, AF-6 seems to be an unique activator of the parkin/PINK1 pathway since it promotes all key steps, from the translocation of parkin to the mitochondria, through parkin ubiquitin-ligase activation to mitochondrial clustering and mitophagy. Thus, AF-6 may be important to maintain proper mitochondrial homeostasis under normal conditions. It is noteworthy that AF-6 soluble levels are decreased in affected PD brain areas, implying that AF-6 deficiency may contribute to mitochondrial dysfunction thought to occur in PD (53). The presence of AF-6 in Lewy bodies indicates that it may be aggregated and inactive at these structures. Like AF-6, the soluble forms of the synphilin-1 isoform, synphilin-1A and USP9X are decreased in α-synucleinopathies (54,55). Thus, it is possible that depletion of critical proteins, like AF-6, from the cytosol of affected neurons may have more implications to the pathogenesis of PD than previously anticipated.

Depolarization by the mitochondrial uncoupler CCCP recruits parkin to the mitochondria, followed by mitochondrial clustering and mitophagy (30,32). Although the real purpose of parkin translocation to the mitochondria is still under debate (56,57), its translocation depends on the presence of PINK1. Depolarization of mitochondria by CCCP stabilizes PINK1 uncleaved form, causing it to stay longer at the membrane, and allowing it to recruit more efficiently parkin to the mitochondria (31). We have shown that AF-6 increases the translocation of parkin to the mitochondria and that both PINK1 overexpression and mitochondrial depolarization by CCCP intensifies this effect. On the other hand, AF-6 is unable to properly activate parkin and PINK1 mutants (29,31,40), indicating that the juvenile disease mutations are insensitive to AF-6. The data are consistent with the notion that AF-6 works in concert with parkin and PINK1 to promote mitochondrial degradation. The finding that AF-6 levels are decreased in PD brains suggests that it may contribute to the mitochondrial dysfunction observed in the disease. Strategies to increase AF-6 levels represent now a tantalizing novel target for future therapeutics.

## MATERIALS AND METHODS

### Materials

[^35^S]Methionine/cysteine was obtained from Perkin-Elmer. Lipofectamine 2000 was purchased from Invitrogen. A complete protease inhibitor cocktail was obtained from Roche. All other reagents were purchased from Sigma.

### Cell culture and transfections

HEK293 and SH-SY5Y cells were grown in DMEM containing 10% fetal bovine serum in a 5% CO_2_ atmosphere. Cells were transiently transfected with *N*-terminal-tagged pRK5 and pFLAG-CMV-2 plasmids utilizing Lipofectamine 2000 and processed after 36 h. For experiments using small-interference RNAs, the cells were transfected with shParkin (5′-GATCCCCGTCACGAAACAAATGCCTCTTCAAGAGAGAGGCATTTGTTTCGTGACTTTTTA-3′; Oligoengine), shAF-6 (5′-CCGGCC AAAGAAATTGCCTGGTGAT CTCGAGATCACCAGGCAATTTCTTTGGTTTTTG-3′; Sigma), siAF-6 (5′-GUAUGCACCUGAUGACAUUdTdT-3′; Ambion) or scrambled shRNA and siRNA controls using Lipofectamine 2000 as previously described (19).

For the shRNA rescue experiments, we generated RNAi-resistant AF-6 and parkin complementary DNAs (cDNAs), including shRNA-resistant myc-AF-6 encoding silent mutations at the codons relative to amino acid residues 1138, 1140 and 1141; siRNA-resistant myc-AF-6 with silent mutations at the codons relative to amino acid residues 842, 843, and 844 as well as shRNA-resistant HA-parkin encoding silent mutations at the codons relative to amino acid residues 78, 79 and 81. To prove the specificity of RNAis used in this study, the RNAi-resistant myc-AF-6 and HA-parkin constructs were co-transfected with their specific RNAis and their ability to rescue from AF-6 and parkin knockdown were investigated accordingly.

### Antibody generation

For anti-AF-6 antibody, rabbits were immunized with the C-terminal amino acids 849–1190 of AF-6 fused to GST. Immune serum was passed through a GST-sepharose 4B column to remove anti-GST antibodies. Pre-cleared serum was incubated for 16 h with GST-AF-6 (849–1190) immobilized on polyvinylidene fluoride strips. Strips were washed with 20 mm Tris pH 7.4 and 500 mm NaCl, and the antibody was eluted from the strips with 100 mm glycine pH 2.5 and dialyzed against phosphate buffered saline (PBS). For anti-parkin antibody, rabbits were immunized with the parkin amino acids 77–237 fused to GST, and the antibody purified as described above.

### Western blot analysis

Samples were homogenized as previously described (58). Blots were probed with the antibodies mouse anti-HA (Covance), rabbit anti-myc, rabbit anti-HA, mouse anti-actin, mouse anti-GAPDH (Santa Cruz), rabbit anti-VDAC (Cell Signaling), mouse anti-myc, rabbit anti-FLAG and rabbit anti-parkin (Sigma). Quantifications of enhanced chemiluminescence reactions were done according to ImageMaster analysis.

For western blot of brain tissues, adult Sprague-Dawley rats were anesthesized with isoflurane and killed by decapitation with the approval of the Technion Animal Experimentation Committee. Brain tissues were homogenized in five volumes/weight of buffer containing 50 mm Tris pH 7.4, 140 mm KCl, 1% of Triton X-100, 30 μm MG132 (Sigma), and protease inhibitor cocktail. Samples were centrifuged at 13 000*g* and supernatants were collected for further analysis by western blot.

Post-mortem human brain tissue from four idiopathic PD and four neurologically normal controls were obtained from the Queen Square Brain Bank archive (55). Appropriate written consent was obtained in all cases and the study was approved by the Local Research Ethics Committee. Brains were processed as previously described (55). Briefly, substantia nigra and caudate plus putamen of sporadic PD and control brains were homogenized in buffer containing 50 mm Tris–HCl, 30 mm NaCl, 30 μm MG132 and protease inhibitor cocktail. The extracts were subsequently clarified by centrifugation at 13 000*g* for 5 min. The samples (50 μg/lane) were resolved on PAGE–SDS gels, and the levels of AF-6 and loading controls were determined by western blot analysis.

### *In vitro* binding assays

Extracts of transfected HEK293 cells were homogenized in 50 mm Tris pH 7.4, 140 mm NaCl, 0.5% Triton X-100, 1% deoxycholate (DOC), 30 µm MG132 and protease inhibitors cocktail. Lysates were clarified by centrifugation at 13 000*g* for 5 min and incubated with GST-fusion protein (5 μg/ml) coupled to glutathione-agarose (Sigma) for 1 h at 4°C. Beads were washed extensively in lysis buffer containing 500 mm NaCl. Bound proteins were analyzed by western blot assays.

### Co-immunoprecipitation assays

For the co-immunoprecipitation of AF-6 with parkin, transfected cells were lysed in buffer containing 50 mm Tris (pH 7.4), 140 mm NaCl, 1% Triton X-100, 1% DOC, 0.1% sodium dodecyl sulphate (SDS), 30 μm MG132 and protease inhibitor cocktail (Complete, Roche). The cell extracts were clarified by centrifugation and incubated for 4 h with anti-HA coupled to protein G beads (Sigma) (58). Immunoprecipitates were washed with lysis buffer containing 500 mm NaCl and detected by western blot.

For endogenous co-immunoprecipitation assays, rat brains were homogenized in buffer containing 50 mm Tris (pH 7.4), 140 mm NaCl, 1% DOC, 1% NP-40, 0.1% SDS, 30 μm MG132 and protease inhibitor cocktail. Brain homogenates were clarified by centrifugation at 13 000*g* for 5 min. Antibody to parkin was coupled to protein G beads (58) and incubated for 7 h with brain homogenate (2 mg/ml). Immunoprecipitates were washed with lysis buffer containing 500 mm NaCl and detected by western blot using an anti-AF-6 antibody.

### *In vivo* ubiquitination assays

Transfected cells were incubated with 10 µm lactacystin for 12 h, lysed in buffer containing 50 mm Tris pH 7.4, 140 mm NaCl and 1% SDS, and immediately boiled at 100°C for 5 min. Lysates were sonicated and diluted ten times with lysis buffer containing 50 mm Tris pH 7.4, 140 mm NaCl, 1% Triton X-100, 30 μM MG132 and protease inhibitor cocktail. Samples were centrifuged at 13 000 g for 5 min and the supernatant was incubated with anti-HA antibody matrix (Sigma) for 4 h at 4°C. Immunoprecipitates were extensively washed with buffer contained 50 mm Tris pH 7.4, 500 mm NaCl, 1% Triton X-100 and 0.1% SDS, and detected by western blot.

### Pulse-chase experiments

Transfected cells were pulsed with methionine/cystein-free medium containing 100 μCi of [^35^S]methionine/cystein (Perkin Elmer) for 6 h and subsequently chased in regular medium for the specified times. The cells were harvested and immunoprecipitation of HA-AF-6 was carried out as described above for the co-immunoprecipitation assays. Immunoprecipitates were resolved on 8% PAGE–SDS gels and the amount of [^35^S] labeled-AF-6 was quantified by PhosphoImager analysis.

### Mitochondrial preparation

Mitochondria were isolated using a discontinuous Percoll gradient as previously described, with a few modifications (59). Briefly, the cells were disrupted using a glass homogenizer in buffer containing 250 mm sucrose, 20 mm Hepes, 3 mm EDTA, 20 mm NaF, 2 mm Na_3_VO_4_, 10 mm PPi, 20 mm β-glycerol phosphate, 30 μm MG132 and protease inhibitor cocktail. The washed mitochondrial pellet was resuspended in a 15% Percoll in cold homogenizing buffer, layered onto a discontinuous Percoll gradient of 23% over 40% and then centrifuged for 20 min at 73 000*g* at 4°C. The interface band between the 23 and 40% Percoll layers was collected and washed twice in cold homogenizing buffer. The mitochondrial pellet was suspended in homogenization buffer and analyzed by western blot assays.

For the *in vivo* ubiquitination experiments using mitochondrial fractions, washed Percoll-purified mitochondria was resuspended in buffer containing 50 mm Tris pH 7.4, 140 mm NaCl, 1% Triton X-100, 0.1% SDS, 30 μM MG132 and protease inhibitor cocktail (Complete, Roche). HA-Parkin from the mitochondrial fraction was immunoprecipitated, washed and analyzed by western blot as described for the *in vivo* ubiquitination assays.

### Immunocytochemistry assays

Transfected SH-SY5Y cells were incubated with or without 10 μm lactacystin, for 12 h, fixed with 4% paraformaldehyde for 15 min and blocked in PBS containing 0.2% Triton X-100 and 5% normal goat serum. The cells were labeled with anti-HA and anti-myc antibodies as previously described (58). Immunolabeling was detected using FITC- and Cy^3^-labeled secondary antibodies (Jackson Laboratories). The percent of cells containing cytosolic inclusions was determined by confocal microscopy in at least 20 fields per experiment.

The mitochondrial co-localization and clustering experiments in SH-SY5Y cells were carried out by co-transfecting GFP-mitochondria (GFP-mito; Clontech). The co-localization of parkin with GFP-mito and the clustering of GFP-mito were determined by confocal microscopy in at least 20 fields per experiment. The mitochondrial clustering experiments in HeLa cells were carried out by co-transfecting stably expressing GFP-parkin HeLa cells, which were then treated with 10 μm CCCP for 1 h and analyzed by confocal microscopy to reveal GFP–parkin clustering at the mitochondria upon CCCP treatment.

For the mitophagy experiments, small amounts of GFP were co-transfected to visualize transfected cells, and mitochondrial presence was determined by staining endogenous Tom20 mitochondrial protein. The percent of cells lacking mitochondria was determined by confocal microscopy in at least 20 fields per experiment.

The statistics for all the confocal analyses mentioned above were calculated using paired Student's *t* test.

### Immunohistochemistry

Immunohistochemistry studies on human brain sections were carried out using paraffin-embedded formalin-fixed sections from four idiopathic PD cases (55). Sections (8 μm thick) were de-waxed and brought to alcoholic phase. Endogenous peroxidase was blocked using 100% methanol containing 0.3% hydrogen peroxide for 10 min followed by thorough washing. Pretreatment to unmask antigenic sites involved pressure cooking the sections in 10 mm citrate buffer for 10 min. Non-specific antigenic sites were blocked using 4% normal goat serum for 30 min at room temperature (RT). The AF-6 antibody (1:500) was incubated at first at RT for 1 h followed by overnight incubation at 4°C. After washing the sections thoroughly, a biotinylated secondary antibody (anti-rabbit broad spectrum antibody Histostain-plus; Zymed) was incubated at RT for 30 min. The sections were then processed by using an ABC-peroxidase kit and diaminobenzidine. The immunolabeled sections were counterstained lightly with Meyers's hematoxylin, dehydrated and coverslip-mounted with di-n-butylphthalate-polystyrene-xylene.

## FUNDING

R.B. is funded by the Reta Lila Weston Institute and is a member of the UKPD consortium. This work was supported by Israel Academy of Sciences, The B. Rappaport Foundation, Dear's Foundation, Braude Foundation Parkinson's Disease Research Fund, A.J. Berman Neurological Research Fund and the Technion Research funds to S.E.
